# Performing photonic nonlinear computations by linear operations in a high-dimensional space

**DOI:** 10.1515/nanoph-2023-0234

**Published:** 2023-06-19

**Authors:** Wenkai Zhang, Wentao Gu, Junwei Cheng, Dongmei Huang, Zihao Cheng, Ping-kong Alexander Wai, Hailong Zhou, Jianji Dong, Xinliang Zhang

**Affiliations:** Wuhan National Laboratory for Optoelectronics, Huazhong University of Science and Technology, 430074 Wuhan, China; Optics Valley Laboratory, 430074 Wuhan, China; The Hong Kong Polytechnic University Shenzhen Research Institute, Shenzhen 518057, China; Department of Electrical Engineering, Photonics Research Institute, The Hong Kong Polytechnic University, Hong Kong, 999077, China; Department of Physics, Hong Kong Baptist University, Kowloon Tong, Hong Kong, 999077, China

**Keywords:** microring resonator, optical digital computing, silicon photonics

## Abstract

As photonic linear computations are diverse and easy to realize while photonic nonlinear computations are relatively limited and difficult, we propose a novel way to perform photonic nonlinear computations by linear operations in a high-dimensional space, which can achieve many nonlinear functions different from existing optical methods. As a practical application, the arbitrary binary nonlinear computations between two Boolean signals are demonstrated to implement a programmable logic array. In the experiment, by programming the high-dimensional photonic matrix multiplier, we execute fourteen different logic operations with only one fixed nonlinear operation. Then the combined logic functions of half-adder and comparator are demonstrated at 10 Gbit/s. Compared with current methods, the proposed scheme simplifies the devices and the nonlinear operations for programmable logic computing. More importantly, nonlinear realization assisted by space transformation offers a new solution for optical digital computing and enriches the diversity of photonic nonlinear computing.

## 1 Introduction

With the rapid development of big data processing and next-generation communications, it is increasingly difficult for conventional electronic hardware to fulfill the skyrocketing demand of computing resources [1, 2]. As a promising alternative to overcome this obstacle, photonic computing has attracted much interest because of its low latency, high frequency, and high parallelism [3, 4]. Among the photonic computation schemes proposed to date, photonic linear operations are quite plentiful and easy to implement by optical elements. Photonic matrix-vector multiplication (MVM), as one of typical linear operations [5–7], can be implemented by various optical elements such as Mach–Zehnder interferometer (MZI) networks [8–10], plane light conversion (PLC) devices [11–13], and microring resonator (MRR) arrays [14–16]. The superiority of ample reconfigurability has also been demonstrated by numerous researches [17–20]. However, when it comes to nonlinear operations like optical digital computing [21–23], the difficulty in the implementation of accurate targeted nonlinear transform increases dramatically, owing to the limited reconfigurability and diversity of photonic nonlinear schemes. For most digital computing researches, the programmable logic computing is especially important because of its flexibility and universality. The existing optical programmable logic array schemes tend to require multiple nonlinear operations and complicated devices [24–27]. As another way of thinking, if the programming ability and diversity of photonic linear computing can be utilized to perform optical nonlinear computing, then accurate implementation of targeted nonlinear computations, such as Boolean logic operations, will be greatly simplified.

In this paper, we present a photonic nonlinear implementation by linear operations in a high-dimensional (HD) space. By utilizing just one unrestricted nonlinear conversion to expand the spatial dimensions, an arbitrary photonic binary nonlinear computation can be accurately executed by performing HD linear photonic transformation. Using Boolean logic as an example, we demonstrate arbitrary logic computations between two binary signals. By programming the photonic MVM, fourteen logic operations of two-input logic are demonstrated with only one fixed nonlinear operation. The combined logic functions of comparator and half-adder are then implemented at 10 Gbit/s. Compared with current methods, the introduction of HD photonic MVM simplifies the devices and the nonlinear operations required to perform programmable logic. Moreover, nonlinear realization assisted by HD linear operations will further extend the scope of photonic nonlinear computing.

## 2 Principle

**Figure 1:**
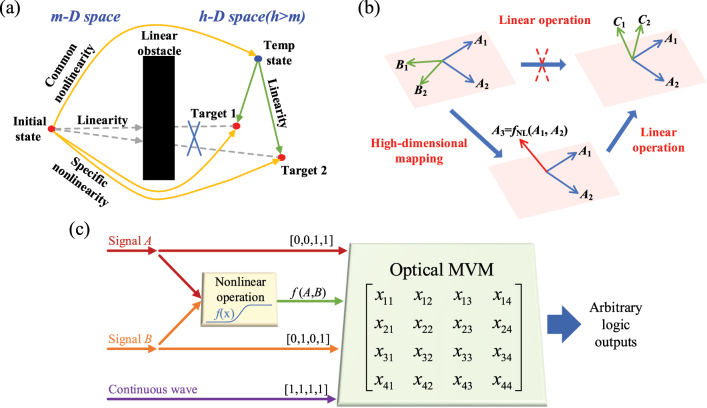
The principle of proposed photonic nonlinear computing. (a) Sketch map of nonlinear computing in the view of spatial dimension. (b) Example of nonlinear computing in a two-dimensional space. (c) Practical application of our proposed concept in Boolean logic operations.

By definition, a nonlinear operation in a linear space cannot be completed directly through any combination of linear operations in the original linear space. To realize specific optical nonlinear functions, most traditional optical methods struggle to find suitable materials and design appropriate structures [28, 29]. It is quite difficult to match the various targets in general, limited by the poor programming ability and lack of diversity of the optical nonlinearity. However, if we can embed the original linear space in a HD space, then the targeted outputs can be achieved by linear operations in the HD space. In other words, the linearity obstacle in the original linear space can be overcome by working in a HD space. As depicted in Figure 1(a), we propose to utilize some common optical nonlinear effects like four-wave mixing (FWM) [30] or easily achievable nonlinear transformations like Ge/Si hybrid structures [31, 32] to map the initial signals (initial state) to a temporary (temp) state in a HD space. Then various nonlinear operations in the original space can be achieved by programming the linear networks in the HD space, to transform the signals from the temp state to the targeted state. For example, the targets (Target 1 and Target 2 in Figure 1(a)) can now be easily achieved by linear conversion only from the temp state.

To explain more clearly, Figure 1(b) shows an example to illustrate the operation principle. The original space is a two-dimensional (2D) plane. The two basis vectors (*A*_1_, *A*_2_) in the 2D plane can only linearly synthesize the output vectors (*B*_1_, *B*_2_) in the original plane. For arbitrary output vectors (*C*_1_, *C*_2_) out of the plane, direct nonlinear mapping from the basis vectors is quite difficult with current optical methods. Our proposed scheme is firstly to generate the new basis vector of *A*_3_ by a nonlinear synthesis from the original basis vectors (*A*_1_, *A*_2_) as

(1) $$A_{3}=f_{NL}\left(A_{1},A_{2}\right),$$

where $f_{NL}\left(A_{1},A_{2}\right)$ is a nonlinear function and the output vector is linearly independent of the original basis vectors. Then a three-dimensional (3D) space can be expressed with the original basis vectors (*A*_1_, *A*_2_) plus the additional basis vector (*A*_3_), and arbitrary output vectors (*C*_1_, *C*_2_) in the 3D space can now be obtained by linear combination of the basis vectors (*A*_1_, *A*_2_, *A*_3_). It is important to note that the nonlinear operation in Eq. (1) does not depend on the output vectors (*C*_1_, *C*_2_). Eq. (1) is used only to generate the basis vectors for the new space. In principle, an arbitrary optical nonlinear effect can be used for the nonlinear synthesis as long as the resulting HD space contains the targeted states. Also, space with other higher dimension can be created by multiple nonlinear syntheses. As a result, arbitrary nonlinear mapping from the original space to the new HD space can then be accomplished by linear operations.

Figure 1(c) shows a practical application of our proposed concept in Boolean logic operations. The output results are nonlinearly related to the input signals for most logic operations. Since there are two input signals but four different input/output states for the two-input logic operations, two new basis vectors are needed to be created from the original input signals (*A*, *B*), in order to construct a four-dimensional (4D) space. Here, one of the two new basis vectors is directly created with a constant continuous wave (*CW*) input, given by [1 1 1 1], and the other one can be created with an optical nonlinear effect between Signal *A* and Signal *B*, such as the nonlinear mapping of the superposed signal by optical four-wave mixing (FWM). In this case, the initial input state with two input vectors is mapped to a temp state with four independent vectors, given by

(2) $$I=\left[\begin{array}{cccc} 0 & 0 & 1 & 1\\ 0 & 1 & 0 & 1 \end{array}\right]\overset{nonlinear\;m\mathrm{appi}ng}{\to }I_{temp}=\left[\begin{array}{cccc} 0 & 0 & 1 & 1\\ f\left(0,0\right) & f\left(0,1\right) & f\left(1,0\right) & f\left(1,1\right)\\ 0 & 1 & 0 & 1\\ 1 & 1 & 1 & 1 \end{array}\right]$$

Here *f*(*A*, *B*) is the response function of nonlinear material. The new matrix *I*_temp_ can span a 4D space, provided that the matrix is with full rank, namely the given operation satisfying the nonlinear mapping condition of *f*(0, 0) + *f*(1, 1) ≠ *f*(0, 1) + *f*(1, 0). Hereafter, the arbitrary logic outputs can be achieved via linear transformation in the 4D space. The linear transformation process performed by optical MVM is expressed as

(3) $$O=T_{\text{MVM}}I_{\text{temp}},$$

where the *I*_temp_ and *O* represent the input and output matrix of the optical MVM, respectively. The transmission matrix *T*_MVM_ of optical MVM is get by

(4) $$T_{\text{MVM}}={OI}_{\text{temp}}^{-1}.$$

Since *I*_temp_ is a full rank matrix, its inverse matrix of $I_{\text{temp}}^{-1}$ exists, meaning *T*_MVM_ can be obtained according to the targeted output matrix. The discussion above infers that arbitrary nonlinear functions between two binary signals can be implemented by using only one fixed nonlinear operation, which has the capacity of realizing two-input programmable logic.

## 3 Results

**Figure 2:**
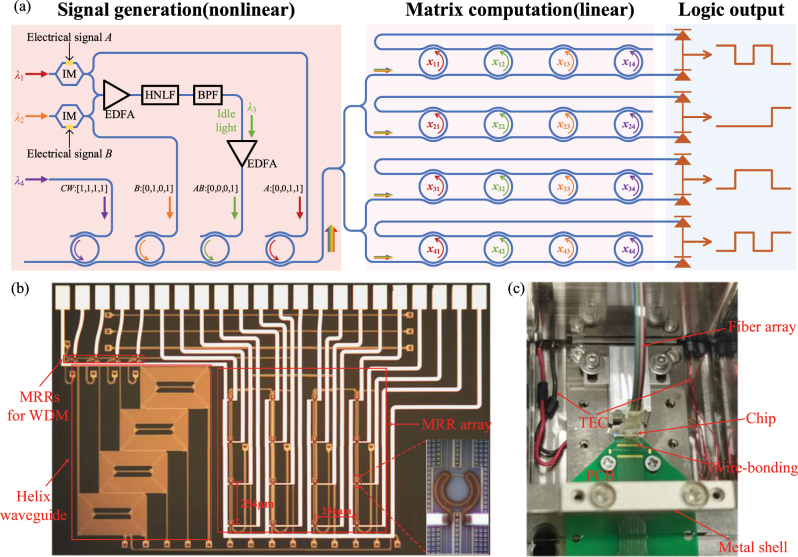
Experimental setup. (a) Schematic diagram of the experimental setup to realize logic computing. (b) Microscope image of the MRR array. (c) Photograph of the packaged chip.

We experimentally demonstrate the HD mapping and linear transformation for logic operations by using a highly nonlinear fiber (HNLF) and an on-chip MRR array, respectively. Figure 2 presents the experimental setup and details of the MRR array chip. The electrical logic Signal *A* and Signal *B* are loaded into the optical carriers at the wavelength *λ*_1_ (1544 nm) and the wavelength *λ*_2_ (1546 nm) respectively by the intensity modulators (IM). Part of them is merged together and incident into the HNLF after amplified by an erbium doped fiber amplifier (EDFA). The idle light at the wavelength *λ*_3_ (1548 nm) will be generated by FWM only when Signal *A* (*λ*_1_) and *B* (*λ*_2_) are input into the HNLF simultaneously, corresponding to both optical signals at high level of 1. Therefore, the signal at *λ*_3_ represents the results of Logic *AND* operation of Signal *A* and Signal *B* [24]. The bandpass filter (BPF) is to select the *AND* signal at *λ*_3_, ensuring the output light only contains the optical signal of Logic *AB*. The following EDFA is to amplify the idle light to the same intensity level of Signal *A* and Signal *B*. The nonlinear operation is written by

(5) $$\left[f\left(0,0\right),f\left(0,1\right),f\left(1,0\right),f\left(1,1\right)\right]=\left[0,0,0,1\right],$$

which satisfies the nonlinear mapping condition of *f*(0, 0) + *f*(1, 1) ≠ *f*(0, 1) + *f*(1, 0). The continuous wave at *λ*_4_ (1550 nm) is also incident into the MRR array, together with the other three beams spanning a 4D space. Hence the targeted logic can now be realized by the linear matrix operation of the MRR array, which can be expressed as

(6) $$\begin{array}{ll} O & =T_{\text{MVM}}I_{\text{temp}}\\ & =\left[\begin{array}{cccc} x_{11} & x_{12} & x_{13} & x_{14}\\ x_{21} & x_{22} & x_{23} & x_{24}\\ x_{31} & x_{32} & x_{33} & x_{34}\\ x_{41} & x_{42} & x_{43} & x_{44} \end{array}\right]\left[\begin{array}{cccc} 0 & 0 & 1 & 1\\ 0 & 0 & 0 & 1\\ 0 & 1 & 0 & 1\\ 1 & 1 & 1 & 1 \end{array}\right], \end{array}$$

where the *x*_*ij*_ is the weight represented by MRR in *i*th row and *j*th column of the MRR array as shown in Figure 2(a).

Figure 2(b) and (c) show the microscope image of the MRR array and the packaged chip. The chip is fabricated on a silicon-on-insulator (SOI) wafer with 220-nm-thick top silicon and 2-μm-thick buried oxide layer. It should be mentioned that we intended to utilize the on-chip helix waveguide to generate the idle light with FWM effect. However, it is experimentally difficult to select the generated idle light by the following MRR filters because the extinction ratio of the MRR is only about 10 dB. As an alternative, we use an off-chip HNLF to execute the nonlinear operation. The MRR filters originally serving as BPFs are now used to combine the four signals at different wavelengths, thus acting as a wavelength division multiplexer (WDM). The mixed optical signals are injected to the MRR array to perform linear transformation. The MRR array contains 16 add-drop MRRs arranged in 4 rows and 4 columns. The radius of MRRs is 10 μm and the adjacent MRRs are separated by a spacing of 254 μm to reduce the thermal crosstalk. By tuning the voltage applied to the surrounding metal electrode of each MRR, the corresponding resonance peak is adjusted to the specific position so that the targeted weight can be performed by the MRR [14, 16]. When the voltages of 16 metal electrodes are set properly, the MRR array will execute an arbitrary 4 × 4 real weight matrix from *−*1 to 1. And the results of matrix computations are then detected by the balanced photodetectors, which can detect the optical power from through and drop ports of MRR array simultaneously and output the power difference of these two ports. (The concrete matrix implementation of the MRR array can be referred in Appendix)

**Figure 3:**
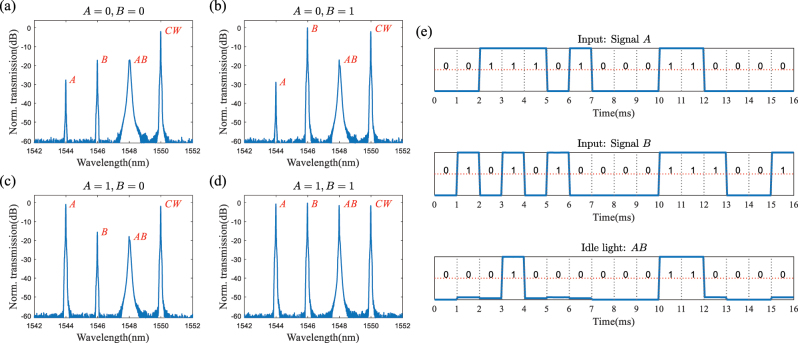
Experimental results of nonlinear synthesis. (a–d) Spectra of nonlinear mapping by FWM for different input states. (e) Output signals of nonlinear synthesis for different input states generated by HNLF.

Figure 3(a–d) depict the experimental output spectra of nonlinear mapping by FWM in different input states. One can see that the idle light at 1548 nm carries the Signal *AB* and is logic 0 as long as either Signal *A* or Signal *B* is at 0, which corresponds to the above-mentioned nonlinear synthesis. Figure 3(e) shows the output waveforms depending on the time-varying input signals, confirming the logic *AND* operation of Signal *A* and Signal *B*. Here, the input Signal *A* and Signal *B* are modulated by IMs at 1 kbit/s. The three signals and the continuous wave are mixed by the MRR-based WDM and input into the MRR array to perform linear transformation.

**Figure 4:**
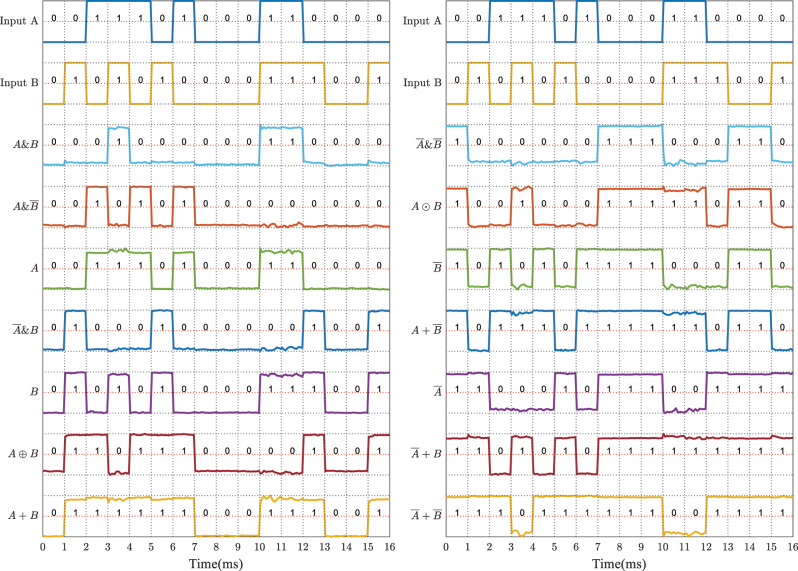
Experimental results for fourteen different logic operations.

By tuning the transmission matrix of the MRR array, arbitrary logic operations can be realized. As shown in Figure 4, fourteen output logic results of two-input logic, except for the outputs of all zeros and all ones, are experimentally demonstrated. Benefited from the reconfigurability and fine-tuning characteristics of the optical MVM, these logic operations are accurately realized via tuning the linear transmission matrix of the optical MVM, and only one fixed nonlinear conversion is required.

**Figure 5:**
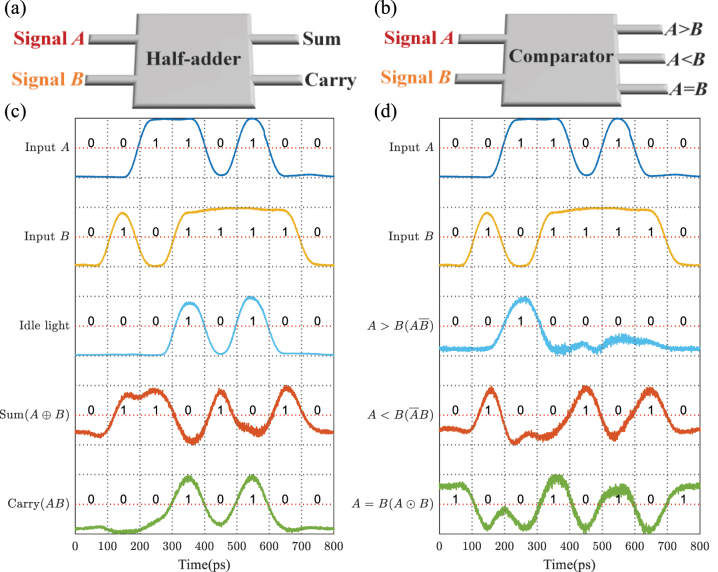
Experimental results for combined logic functions. The structure diagrams of (a) half-adder and (b) comparator. (c) Waveforms of input optical Signal *A* and Signal *B*, the idle light generated by HNLF, and the half-adder. (d) Waveforms of input Signal *A* and Signal *B*, and the comparator.

Considering the given MRR array has multiple output ports, combined logic functions can be realized by outputting logic results at different ports simultaneously. Here, we control the MRR array to execute some advanced functions, such as half-adder and comparator. Figure 5(a) and (b) present the structure diagrams, respectively. And the truth table of these logic functions is shown in Table 1.

**Table 1:** Truth table of the half-adder and the comparator.

Half-adder				Comparator					
*A*	*B*	*A* ⊕ *B*(Sum)	*AB*(Carry)	*A*	*B*	State	$A\bar{B}$	$\bar{A}B$	*A* ⊙*B*
0	0	0	0	0	0	*A* = *B*	0	0	1
0	1	1	0	0	1	*A* < *B*	0	1	0
1	0	1	0	1	0	*A* > *B*	1	0	0
1	1	0	1	1	1	*A* = *B*	0	0	1

Figure 5(c) shows the input optical Signal *A* and Signal *B*, the signal of the idle light, and the output logic results of half-adder at 10 Gbit/s. Then we change the matrix represented by the MRR array to perform the function of comparator. Figure 5(d) depicts the experimental results. The output waveforms of both half-adder and comparator are performed correctly according to the input waveforms and the truth table. Note that here we just verify the MRR array’s capacity of parallel outputs. Other functions like 2–4 decoder and 1–2 data distributor can also be realized via tuning the MRR array to an appropriate transmission matrix.

## 4 Discussion

Here, the MRR-MVM and FWM effect are used to demonstrate principle of photonic nonlinear computations. Note that the proposed realization of logic computation is not confined to the optical devices used in this work. There is no need to search for suitable nonlinear materials or design specific structures to fit the corresponding logic operations. As long as the four input states are set appropriately according to the nonlinear mapping condition, other nonlinear schemes are also available, such as graphene/Si waveguide [33], germanium silicon hybrid coupler [32] and cavity-loaded Mach–Zehnder interferometer [34]. The on-chip nonlinear methods make the coherent computing possible. In this case, we can utilize MZI networks to perform linear transformation with a larger bandwidth to support higher computation speed.

It should be mentioned that the proposed method is not limited to 2-bit input. We give the nonlinear demonstration of 3-bit input scenario and then expand it to *N*-bit. The three input signals can be defined as *A*, *B*, and *C*, and we use the logic *AND* as the nonlinear operation to create new spatial bases. The five additional bases can be given by *AB*, *AC*, *BC*, *ABC*, and the *CW* input, expressed as:

(7) $$\begin{array}{ll} I_{\text{input}} & =\left[\begin{array}{cccccccc} 0 & 0 & 0 & 0 & 1 & 1 & 1 & 1\\ 0 & 0 & 1 & 1 & 0 & 0 & 1 & 1\\ 0 & 1 & 0 & 1 & 0 & 1 & 0 & 1 \end{array}\right]\overset{\begin{array}{c} nonlinear\\ mapping \end{array}}{\to }I_{\text{temp}}\\ & =\left[\begin{array}{ccccccccc} A: & 0 & 0 & 0 & 0 & 1 & 1 & 1 & 1\\ B: & 0 & 0 & 1 & 1 & 0 & 0 & 1 & 1\\ C: & 0 & 1 & 0 & 1 & 0 & 1 & 0 & 1\\ AB: & 0 & 0 & 0 & 0 & 0 & 0 & 1 & 1\\ AC: & 0 & 0 & 0 & 0 & 0 & 1 & 0 & 1\\ BC: & 0 & 0 & 0 & 1 & 0 & 0 & 0 & 1\\ ABC: & 0 & 0 & 0 & 0 & 0 & 0 & 0 & 1\\ CW: & 1 & 1 & 1 & 1 & 1 & 1 & 1 & 1 \end{array}\right]. \end{array}$$

where the eight bases are linearly independent apparently, which means the *I*_temp_ is a full rank matrix, and thus 3-bit input arbitrary NFs can be realized. For *N*-bit input, we can select two, three, four, …, *N* of them to perform logic *AND* operations, whose results are linearly independent. And the number of bases *N*_*b*_ is given as:

(8) $$N_{b}=\overset{N}{\underset{i=2}{\sum }}C_{N}^{i}+N+1=2^{N},$$

where $\overset{N}{\underset{i=2}{\sum }}C_{N}^{i}$ is the number of bases created by logic *AND* operations, *N* is the number of input signals, and the additional basis is the *CW*. All of these bases are linearly independent and will construct a 2^*N*^-dimendional space, which is capable of performing any nonlinear operations of *N*-bit input with the following linear transformation.

## 5 Conclusions

In conclusion, we propose a novel method to perform photonic nonlinear computations by linear operations in a HD space and apply the principle to realize all-optical programmable logic array. By employing an MRR array to execute the MVM, the programmable logic operations are realized with only one fixed nonlinear operation using HNLF. Fourteen two-input logic operations have been demonstrated at 1 kbit/s. We also realize the combined logic functions of half-adder and comparator at a high speed of 10 Gbit/s. Compared with current methods, the proposed scheme simplifies the devices and the nonlinear operations required to perform programmable logic. And the model is extended to *N* input binary signals. The application of the proposed scheme is not confined to Boolean logic; it can also support arbitrary nonlinear functions between *N* binary signals. The implementation of nonlinear operation by introduction of linear transformation offers more possibility for optical nonlinear realization and extends the use of existing photonic nonlinearity.

## Figures and Tables

**Figure S1:**
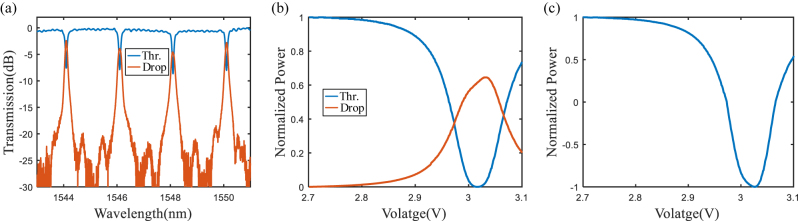
Matrix implementation of the MRR array. (a) The spectrum of the MRR array when each MRR resonates at the corresponding input wavelength. (b) The normalized optical power of the through port and drop port of a certain MRR. (c) The normalized optical power after difference calculation.

## Appendix

### Matrix implementation of the MRR array

To get the targeted matrix of the MRR array, we first tune the voltages of MRR electrodes to make MRRs resonate at the corresponding input wavelengths (the weight *x*_*ij*_ = −1) shown in Figure S1(a). For each MRR, we reduce the voltage of the MRR electrode so that the MRR is out of resonance (*x*_*ij*_ = 1), then increase the voltage at the step of 1 mV, applying at least 400 voltage steps to the MRR electrodes (different from the specific MRR, if the resonance voltage is less than 3 V, more voltage steps are required to cover the weight *x*_*ij*_ from −1 to 1), and measure the output power of the through port and drop port. Figure S1(b) and (c) depict the relation between voltage and transmission of the MRR, according to which we can tune the MRR to perform targeted weight by loading the corresponding voltage to the MRR electrode. The matrix performed by the MRR array has about 5-bit tuning accuracy, which is sufficient for the logic binary output. Note that the maximum power of through port is usually higher than drop port. For this case, an optical attenuator can be added following the through port to balance the maximum power. After all electrodes are properly adjusted, the desired matrix can be implemented by the MRR array.
